# Detection of *Streptococcus pneumoniae* in sterile liquids using real-time PCR (qPCR) in hospitalized patients with suspected invasive pneumococcal disease

**DOI:** 10.17843/rpmesp.2025.421.14390

**Published:** 2025-03-18

**Authors:** Brayan E. Gonzales, Erik H. Mercado, Marcela Lopez-Briceño, David Durand Vara, Francisco Campos, Eduardo Chaparro, Olguita Del Águila, María E. Castillo, Andrés Saenz, Isabel Reyes, Roger Hernandez, Theresa J. Ochoa

**Affiliations:** 1 Alexander von Humboldt Institute of Tropical Medicine, Cayetano Heredia Peruvian University, Lima, Peru. Cayetano Heredia Peruvian Universit Alexander von Humboldt Institute of Tropical Medicine Cayetano Heredia Peruvian Universit Lima Peru; 2 Peruvian Pneumococcal Research Group (GPIN), Lima, Peru. Peruvian Pneumococcal Research Group (GPIN) Lima Peru; 3 Department of Pediatrics, Madre-Niño San Bartolomé National Teaching Hospital, Lima, Peru. Department of Pediatrics Madre-Niño San Bartolomé National Teaching Hospital Lima Peru; 4 Faculty of Medicine, Cayetano Heredia Peruvian University, Lima, Peru. Cayetano Heredia Peruvian University Faculty of Medicine Cayetano Heredia Peruvian University Lima Peru; 5 Department of Pediatrics, Cayetano Heredia National Hospital, Lima, Peru. Department of Pediatrics Cayetano Heredia National Hospital Lima Peru; 6 Clinical Specialty Pediatrics Service, Edgardo Rebagliati Martins National Hospital, Lima, Peru. Clinical Specialty Pediatrics Service Edgardo Rebagliati Martins National Hospital Lima Peru; 7 Epidemiology Office, National Institute of Child Health, Lima, Peru. Epidemiology Office National Institute of Child Health Lima Peru; 8 Department of Pediatrics, Daniel Alcides Carrión National Hospital, Lima, Peru. Department of Pediatrics Daniel Alcides Carrión National Hospital Lima Peru; 9 Hospitalization Service, Pediatric Emergency Hospital, Lima, Peru. Hospitalization Service Pediatric Emergency Hospital Lima Peru

**Keywords:** Streptococcus pneumoniae, qPCR, invasive pneumococcal disease, pneumoniae, meningitis, molecular methods

## Abstract

The standard for diagnosing invasive pneumococcal disease (IPD) is to isolate pneumococcus in culture. However, the etiological agent cannot be identified in some patients, especially those who received empirical antibiotic therapy. This study aimed to detect pneumococcus in normally sterile fluids by qPCR in patients with suspected IPD hospitalized in Lima. qPCR had a detection limit of 1.2 x 101 genome copies/uL. Of the 71 clinical samples (51 were pleural fluid [PF] and 20 were cerebrospinal fluid [CSF]), 29.4% (28/71) were positive for pneumococcus by culture and 71.8% (51/71) were positive by qPCR, including 78.4% (40/51) in PF and 55.0% (11/20) in CSF. Of the positive samples, 13/51 were serotype 19A. The detection of pneumococcus was almost double by qPCR compared to the conventional microbiological method. Therefore, molecular methods such as qPCR should be implemented to improve the identification and timely treatment of IPD in Peru and in the region.

## INTRODUCTION

*Streptococcus pneumoniae* (pneumococcus) is a Gram-positive, encapsulated diplococcus with more than 100 serotypes that causes high morbidity and mortality rates, particularly in children, older adults, and patients with chronic diseases in low- and middle-income countries ^1^. Pneumococcus causes non-invasive disease (otitis or sinusitis) and invasive pneumococcal disease (IPD) such as bacteremia, pneumonia, and meningitis. It is one of the leading causes of acute otitis media, pneumonia, and bacterial meningitis in children after the introduction of the *Haemophilus influenzae* type b vaccine worldwide ^2^. Although the incidence of IPD has increased in the last two decades, it is expected to decline with the widespread use of pneumococcal conjugate vaccines (PCV) ^3^.

Microbiological culture is the primary method for detecting *S. pneumoniae* and diagnosing IPD, but it often yields negative results in patients who have received antibiotics prior to sample collection. The use of BioFire® FilmArray®Panels offers high accuracy in pathogen detection, but they are expensive and difficult to access for many healthcare centers ^4^. Because of this, the use of more accessible molecular tests is recommended, such as conventional PCR or real-time PCR (qPCR), which offer results comparable to automated methods ^5^.

Given this situation, there is a need to evaluate usually-sterile fluids using molecular methods to optimize diagnosis. Molecular methods such as qPCR are used in blood, pleural fluid (PF), and cerebrospinal fluid (CSF) samples ^6^^,^^7^. The sensitivity and specificity of these methods are higher than those of microbiological methods ^8^ and represent a promising strategy for identifying these pathogens in patients with clinical suspicion of IPD.

In the absence of local data on the use of molecular methods in clinical samples, we conducted this study in order to determine pneumococcus in normally sterile fluids using qPCR in hospitalized patients with suspected IPD in national hospitals and private clinics in Lima, Peru, between 2016 and 2023.

**KEY MESSAGES**

Motivation for the study. Invasive pneumococcal disease (IPD) is usually diagnosed by microbiological culture to detect pneumococcus. However, this is sometimes not possible, particularly in patients who have previously received antibiotics. This study sought to detect pneumococcus using a molecular technique such as qPCR in hospitalized patients in Lima with suspected IPD.

Main findings. qPCR detected a higher frequency of pneumococcus than the standard microbiological technique.

Implications for public health. These findings suggest that the implementation of qPCR could significantly improve the identification and treatment of IPD in Peru.

## THE STUDY

### Study design

Multicenter cross-sectional case series study of IPD in seven national hospitals (2 de Mayo National Hospital, Cayetano Heredia National Hospital, National Pediatric Emergency Hospital, Daniel Alcides Carrión National Hospital, Edgardo Rebagliati Martins National Hospital, San Bartolomé Mother and Child Teaching Hospital, and the National Institute of Child Health in Breña) and seven clinics and private laboratories (Centenario Clinic, International Clinic, Anglo-American Clinic, Delgado Clinic, Good Hope Clinic, ROE Laboratories, and Medlab Laboratories) in Lima, Peru, between 2016 and 2023.

### Study population

Hospitalized patients of all ages with suspected pneumonia (with pleural effusion or empyema) or bacterial meningitis with samples of normally sterile fluids (pleural fluid and CSF) that were collected by the treating physician during hospital care for diagnostic purposes and that met the case definition.

### Case definition

Invasive pneumococcal disease: clinical suspicion of pneumococcal infection in patients with meningitis, pneumonia with effusion or empyema, bacteremia, septic arthritis, and bacterial peritonitis. For this study, we only included patients diagnosed with community-acquired pneumonia with parapneumonic effusion or empyema and bacterial meningitis with a positive or negative microbiological result for pneumococcus performed at the participating hospital or clinic.

Community-acquired pneumonia: lung infection, usually within seven days, with symptoms and signs of respiratory distress (fever, tachypnea, hypoxemia, dyspnea, pleuritic pain, cough, mucopurulent sputum production, signs of consolidation or effusion such as dullness, crackles, decreased breath sounds), and evidence of pulmonary consolidation on chest X-ray.

Parapneumonic effusion: presence of fluid in the pleural space ≥5 cm on chest X-ray or chest ultrasound with characteristics of exudate [pleural fluid protein/blood protein ratio >0.5, lactate dehydrogenase (LDH) pleural fluid/blood LDH ratio >0.6, pleural fluid LDH >2/3 upper normal limit in blood].

Empyema: Exudate in the pleural space with the presence of pus and/or Gram stain or positive culture in pleural fluid.

Bacterial meningitis: inflammation of the meninges, with signs and symptoms of fever, neck stiffness, altered level of consciousness, and headache, as well as CSF with leukocytes ≥10 cells/mm^3^ with a predominance of polymorphonuclear cells, proteins >40 mg/dL, glucose <40 mg/dL, or CSF/blood glucose ratio <0.5 ^9^.

### Clinical data

Basic demographic and clinical data were collected from medical records, including age, sex, day of sample collection after hospitalization, previous use of antibiotics (before culture), and culture results.

### Laboratory study

PF and CSF samples collected at hospitals or clinics were transported to the Pediatric Infectious Diseases Laboratory at Cayetano Heredia Peruvian University (UPCH), where they were stored at -20°C until processing. DNA extraction was performed with 200 µL of sample using the High Pure PCR Template Preparation Kit® (Roche, Switzerland). For the molecular diagnosis of *S. pneumoniae*, a SYBR Green-based qPCR (SsoAdvancedTM Universal SYBR® Green Supermix, BioRad) was standardized using primers that amplified the lytA gene encoding autolysin in *S. pneumoniae*, which is a methodology previously described and validated and is not part of a commercial kit for the detection of this pathogen ^10^^,^^11^. The CFX96TM Real-Time System (Bio-Rad®, USA) was used with initial denaturation conditions of 95°C for 2 min, with 40 amplification cycles with denaturation at 95°C for 15 sec, hybridization at 60°C for 30 sec, and final extension at 95°C for 30 sec, in addition to a melting curve between 70-95°C with an increase of 0.5 every 0.5 sec. Pneumococcal serotyping was performed in samples positive for the *lytA* gene using a conventional sequential multiplex PCR system developed by the Streptococcus Laboratory (StrepLab) of the CDC-USA ^12^.

The standard strain of *S. pneumoniae* serotype 2 (D39/NCTC7466) was used to determine the detection limit of qPCR. Serial dilutions were conducted in a factor of 10 in the range of 1.2 x 10^6^ to 1.2 x 10^0^ genome copies/uL. The test efficiency value was determined by generating a linear regression evaluating the slope value, linearity correlation coefficient (R^2^), and amplification efficiency.

### Statistical analysis

The characteristics were described and frequencies were compared using the Chi-square test and Fisher’s exact test in the statistical program Stata/SE v.18.0. A p-value of <0.05 was considered statistically significant.

### Ethical considerations

The study was registered in the Decentralized Research Information and Monitoring System (SIDISI 100703) and evaluated by the Institutional Ethics Committee of the Cayetano Heredia Peruvian University (certificate 062-02-22) and by each participating hospital and clinic. Informed consent was requested from patients or parents of pediatric patients to use remnants of PF and CSF samples previously taken by the treating physician for diagnostic purposes.

## FINDINGS

A total of 71 fluid samples (51 PF and 20 CSF) were analyzed from patients with suspected IPD, according to the case definition. Of these patients, 53.5% were male with a median age of 3 years (IQR: 1-9), and 93.9% of cases were children. We found that 76.6% of samples were collected within the first 7 days of hospitalization, and 63.6% received antibiotics prior to culture (Table 1).

**Table 1:** Characteristics of hospitalized patients with meningitis or pleural effusion (N=71).

Characteristics		n (%)
Sex, male		38/71 (53.5)
Age (months) ^a^		3 (1 - 9)
Age group		
	Breastfeeding infants (<2 years)	18 (25.7)
	Preschoolers (2-6 years old)	28 (40.0)
	Schoolchildren (6 - <18 years old)	19 (27.2)
	Adults (≥18 years)	5 (7.1)
Days from hospitalization to sample collection		
	0 - ≤3	32 (50.0)
	>3 - ≤7	17 (26.6)
	>7	15 (23.4)
Received antibiotics prior to culture		42/66 (63.6)
Sample type		
	Pleural fluid	51 (71.8)
	Cerebrospinal fluid	20 (28.2)
Culture result		
	*Streptococcus pneumoniae*	28 (39.4)
	Negative	38 (53.5)
	Other ^b^	5 (7.1)
qPCR *lytA,* positive		51/71 (71.8)
*Cycle threshold* (CT)		
	<25	23 (45.1)
	≥25 - <30	10 (19.6)
	≥30	18 (35.3)
Serotypes ^c^		
	Not determined (low load)	28
	19A	13
	23A	2
	Serogroup 24	2
	10A	1
	Serogroup 6	1
	Not classifiable	4

a Median (interquartile range).

b *Staphylococcus aureus*, *Streptococcus group* B, *Staphylococcus epidermidis*.

c Serotype determined by sequential multiplex PCR.

**Figure 1:**
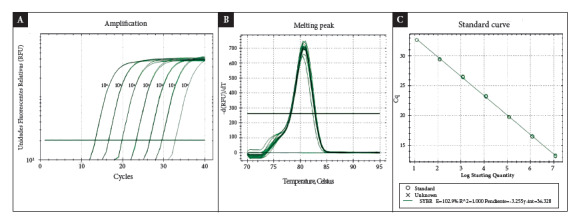
Concentration curve of *S. pneumoniae* using qPCR. A. Evaluation of qPCR using different concentrations of DNA showed that the detection limit is 1.2 x 10^1^ genome copies/uL. B. The melting curves of DNA extracted from the standard strain overlap, demonstrating the reproducibility of qPCR. C. qPCR showed an efficiency of 102.9% for the detection of the *lytA* gene with a correlation coefficient of 1.0.

The detection limit of qPCR was 1.2 x 10^1^ genome copies/uL, with 40 amplification cycles, and no non-specificities were reported after this amplification cycle. qPCR showed an amplification efficiency value of 102.9% and a melting temperature of the *lytA* gene of 80.0 ± 0.50 °C (Figure 1).

Of the total positive samples by qPCR, 23 (45.1%) had an amplification threshold or cycle threshold (Ct) <25, i.e., a high bacterial load; the serotype was determined in these samples, with 19A being the most frequent, found in 13 samples (25.5% of the total) (Table 1). The serotype could not be determined due to low bacterial load in the remaining samples (CT≥25).

*S. pneumoniae* was detected by qPCR in 51 (71.8%) of the samples analyzed, including 78.4% (40/51) in PF and 55.0% (11/20) in CSF (Table 2). Of these samples, only 28 (39.4%) were positive for *S. pneumoniae* by culture. On the other hand, of the 38 culture-negative samples, *S. pneumoniae* was detected in 20 (52.6%) by qPCR. Pneumococcus was identified by qPCR in 3 of 5 samples in which another pathogen was isolated by culture, suggesting coinfection. Pneumococcus detection was higher when the sample was collected in the first days of hospitalization; however, the difference was not significant with late samples. In 42 samples collected after the start of antibiotics, pneumococcus was detected in 20 (47.6%) by culture vs. 33 (78.6%) by qPCR.

**Table 2:** qPCR results for the diagnosis of *S. pneumoniae* in normally sterile fluids according to sample characteristics (N=71) ^a^.

Characteristics		N	**qPCR *lytA* positive**	Positive pneumococcal culture
			51/71	28/71
			n (%)	n (%)
qPCR *lytA*				
	Negative	20	0 (0.0)	0 (0.0)
	Positive	51	51 (71.8)	28 (54.9)
Diagnosis by culture				
	Negative	38	20 (52.6)	0 (0.0)
	*S. pneumoniae*	28	28 (100.0)	28 (39.4)
	Other ^b^	5	3 (60.0)	0 (0.0)
Type of fluid				
	Pleural fluid	51	40 (78.4)	21 (41.2)
	Cerebrospinal fluid	20	11 (55.0)	7 (35.0)
Days from hospitalization to sample collection				
	0 - ≤3	32	26 (81.3)	16 (50.0)
	>3 - ≤7	17	13 (76.5)	7 (41.2)
	>7	15	9 (60.0)	4 (26.7)
Received antibiotics prior to culture				
	No	24	15 (62.5)	7 (29.2)
	Yes	42	33 (78.6)	20 (47.6)

a Some variables may total less than 71 due to missing data.

b *Staphylococcus aureus, Streptococcus group* B, *Staphylococcus epidermidis*.

## DISCUSSION

Our results show that pneumococcal detection in normally sterile fluids in patients with suspected IPD increased from 39.4% by culture to 71.8% by qPCR. Positivity was 78.4% in PF and 55.0% in CSF. Overall, positivity was higher when the sample was taken within the first three days of hospitalization (81.3%). Previous antibiotic use did not affect pneumococcal detection by qPCR.

An important fact is that 92.8% of the samples were from pediatric patients. More than ten years after the introduction of pneumococcal conjugate vaccines (PCV) into Peru’s national immunization program, cases of IPD have decreased ^13^. However, these data show that the pediatric population remains the most affected by this condition.

Multiple studies report the ability of molecular techniques to improve pathogen detection compared to microbiological techniques using normally sterile fluid samples. In the case of patients with meningitis, reports such as those from Sao Paulo, Brazil, show that when qPCR was used, the positivity rate for *S. pneumoniae* increased from 9.9% to 14.4% in 263 CSF samples ^14^. A similar increase in positivity was reported in a study of Mexican patients, in which it increased from 0.9% to 5.1% in 512 CSF samples ^5^. In patients with suspected meningitis in Morocco, the use of qPCR increased positivity from 11% to 19% ^15^. In a study in Fiji in 17 patients with pneumococcal meningitis confirmed by several methods (Gram stain, culture, latex agglutination, and qPCR), culture positivity was 41.0% (7/17), while qPCR positivity was 100.0% (16/16) ^16^. Similarly, in a study conducted in Egypt, 28% of 50 CSF samples were positive for *S. pneumoniae* by microbiology and 52% by qPCR ^17^.

Regarding the detection of *S. pneumoniae* in pleural fluid samples, our results show a 41.2% positivity rate using microbiology and a 78.4% positivity rate using qPCR. In a study evaluating 60 pleural fluid samples confirmed with pneumococcal infection through 16S and/or PCR, only 6 (10%) had a positive culture, while 54 (90%) were positive by PCR ^18^. Similarly, in a quasi-experimental study in the United States, where children diagnosed with complicated pneumonia were evaluated before and after the implementation of *S. pneumoniae* detection by qPCR in pleural fluid, *S. pneumoniae* detection increased by 34.5% after the implementation of qPCR, which complements the microbiological culture previously used in that study ^8^. These findings suggest that the improvement in detection by PCR compared to microbiological culture are not related to a single type of biological sample.

The length of hospital stay and antibiotic use prior to sampling were not associated with lower qPCR positivity for the diagnosis of *S. pneumoniae*. In contrast, a cross-sectional study of 4,676 pediatric and adult patients diagnosed with community-acquired pneumonia reported that, in both blood cultures and sputum cultures, bacterial detection was 2.6% (p<0.01) and 23.2% (p<0.01) higher when samples were collected prior to antibiotic use. However, this did not affect PCR detection or the urine pneumococcal antigen test ^19^. These reports indicate that the use of empirical antibiotic therapy from hospital admission in cases of meningitis and pneumonia could affect the culture of microorganisms using standard microbiological methods.

One limitation of our study was that only the *lyta* gene was used as a target for detecting *S. pneumoniae*. This gene can also be found in other bacteria such as *Streptococcus pseudoneumoniae* and mitis group streptococci ^20^. New recommendations suggest combining genes such as *plyA* or *psaA* for pneumococcal detection by qPCR ^18^. However, this recommendation is for studies in carriers, since *S. pneumoniae* shares the colonization site with other microorganisms. Despite this, qPCR that detects the *lytA* gene remains one of the most sensitive methods for detecting invasive pneumococcal disease ^5^.

In conclusion, standardization of qPCR with a detection limit of 1.2x10^1^ genome copies/uL is a useful tool for improving pneumococcal detection in normally sterile fluids with suspected IPD. qPCR represents a rapid alternative to the three-day wait for culture results, allowing early initiation of appropriate antibiotic therapy. This technique is also useful in cases of prior antibiotic use.
