# Supplementary material for: Detection of Streptococcus pneumoniae in sterile liquids using real-time PCR (qPCR) in hospitalized patients with suspected invasive pneumococcal disease
Source: Rev Peru Med Exp Salud Publica. 2025 Mar 18;42(1):70–5. doi: 10.17843/rpmesp.2025.421.14390 (PMC12176015; doi:10.17843/rpmesp.2025.421.14390)
Supplement: Translation

# Translation: Detección de *Streptococcus pneumoniae* en líquidos estériles mediante una PCR en tiempo real (qPCR) en pacientes hospitalizados con sospecha de enfermedad neumocócica invasiva

**Authors:** Brayan E. Gonzales, Erik H. Mercado, Marcela Lopez-Briceño, David Durand Vara, Francisco Campos, Eduardo Chaparro, Olguita Del Águila, María E. Castillo, Andrés Saenz, Isabel Reyes, Roger Hernandez, Theresa J. Ochoa

**Affiliations:** 1 Instituto de Medicina Tropical Alexander von Humboldt, Universidad Peruana Cayetano Heredia, Lima, Perú.; 2 Grupo Peruano de Investigación en Neumococo (GPIN), Lima, Perú.; 3 Departamento de Pediatría, Hospital Nacional Docente Madre-Niño San Bartolomé, Lima, Perú.; 4 Facultad de Medicina, Universidad Peruana Cayetano Heredia, Lima, Perú.; 5 Departamento de Pediatría, Hospital Nacional Cayetano Heredia, Lima, Perú.; 6 Servicio de Pediatría de Especialidades Clínicas, Hospital Nacional Edgardo Rebagliati Martins, Lima, Perú.; 7 Oficina de Epidemiología, Instituto Nacional de Salud del Niño, Lima, Perú.; 8 Departamento de Pediatría, Hospital Nacional Daniel Alcides Carrión, Lima, Perú.; 9 Servicio de Hospitalización, Hospital de Emergencias Pediátricas, Lima, Perú.

**Keywords:** Streptococcus pneumoniae, qPCR, enfermedad neumocócica invasiva, neumonía, meningitis, métodos moleculares

## Abstract

El estándar para diagnosticar enfermedad neumocócica invasiva (ENI) es aislar neumococo en cultivo. Sin embargo, en algunos pacientes, sobre todo los que recibieron terapia antibiótica empírica, no se logra identificar el agente etiológico. El objetivo de este estudio fue detectar neumococo en líquidos normalmente estériles mediante qPCR en pacientes con sospecha de ENI hospitalizados en Lima. La qPCR tuvo un límite de detección de 1,2 x 10^1^ copias del genoma/uL. De las 71 muestras clínicas (51 de líquido pleural [LP] y 20 de líquido cefalorraquídeo [LCR]), el 29,4% (28/71) fueron positivas para neumococo por cultivo y 71,8% (51/71) fueron positivas por qPCR, incluyendo 78,4% (40/51) en LP y 55,0% (11/20) en LCR. De las muestras positivas, 13/51 fueron del serotipo 19A. La detección de neumococo fue casi el doble por qPCR en comparación con el método microbiológico convencional. Por lo tanto, se debe implementar métodos moleculares como esta qPCR para mejorar la identificación y tratamiento oportuno de ENI en Perú y en la región.

## INTRODUCCIÓN

*Streptococcus pneumoniae* (neumococo) es un diplococo grampositivo, capsulado, con más de 100 serotipos, que causa alta morbilidad y mortalidad, especialmente en niños, adultos mayores, pacientes con enfermedades crónicas en países de medianos y bajos ingresos ^1^. Neumococo causa enfermedad no invasiva (otitis o sinusitis) y enfermedad neumocócica invasiva (ENI) como bacteriemia, neumonía y meningitis. Es una de las principales causas de otitis media aguda, neumonía y meningitis bacteriana en niños después de la introducción de la vacuna contra *Haemophilus influenzae* tipo b en el mundo ^2^. Aunque la incidencia de ENI aumentó en las últimas dos décadas, se espera que disminuya con el uso generalizado de vacunas conjugadas neumocócicas (PCV, por sus siglas en inglés) ^3^.

El cultivo microbiológico es el método principal para detectar S. *pneumoniae* y diagnosticar ENI, pero a menudo resulta negativo en pacientes que han recibido antibióticos antes de la toma de muestra. El uso de BioFire® FilmArray®Panels ofrecen alta precisión en la detección de patógenos, pero son costosos y de difícil acceso en muchos centros de salud ^4^. Debido a esto, se recomienda el uso de pruebas moleculares más accesibles, como la PCR-convencional o en tiempo real (qPCR), que ofrecen resultados comparables a los métodos automatizados ^5^.

Ante esta situación, surge la necesidad de evaluar los líquidos provenientes de sitios normalmente estériles por métodos moleculares para optimizar el diagnóstico. Los métodos moleculares como la qPCR son empleados en muestras de sangre, líquido pleural (LP), líquido cefalorraquídeo (LCR) ^6^^,^^7^. La sensibilidad y especificidad de estos métodos son mayores en comparación a los métodos microbiológicos ^8^^)^ y representan una estrategia prometedora para identificar estos patógenos en pacientes con sospecha clínica de ENI.

Al no tener datos locales sobre el uso de métodos moleculares en muestras clínicas, realizamos este estudio con el objetivo de determinar neumococo en líquidos normalmente estériles mediante qPCR en pacientes hospitalizados con sospecha de ENI en hospitales nacionales y clínicas privadas en Lima, Perú entre 2016 y 2023.

**MENSAJE CLAVE**

Motivación para realizar el estudio. El diagnóstico de la enfermedad neumocócica invasiva (ENI) normalmente se hace mediante cultivo microbiológico para detectar a neumococo. Sin embargo, a veces esto no es posible, especialmente en pacientes que han recibido antibióticos antes. Este estudio buscó detectar neumococo usando una técnica molecular como la qPCR en pacientes hospitalizados en Lima con sospecha de ENI.

Principales hallazgos. La qPCR detectó una mayor frecuencia de neumococo que la técnica microbiológica estándar.

Implicancias para la salud pública. Estos hallazgos sugieren que la implementación qPCR podría mejorar de manera significativa la identificación y tratamiento de ENI en Perú.

## EL ESTUDIO

### Diseño de estudio

Estudio transversal multicéntrico de serie de casos de ENI en siete hospitales nacionales (Hospital Nacional 2 de Mayo, Hospital Nacional Cayetano Heredia, Hospital Nacional Emergencias Pediátricas, Hospital Nacional Daniel Alcides Carrión, Hospital Nacional Edgardo Rebagliati Martins, Hospital Docente Madre-niño San Bartolomé y Instituto Nacional de Salud del Niño Sede Breña) y siete clínicas, y laboratorios privados (Clínica Centenario, Clínica Internacional, Clínica Angloamericana, Clínica Delgado, Clínica Good Hope, Laboratorios ROE y Laboratorios Medlab) en Lima, Perú entre 2016 y 2023.

### Población de estudio

Pacientes hospitalizados de todas las edades con sospecha de neumonía (con derrame pleural o empiema) o meningitis bacteriana con muestras de líquidos normalmente estériles (LP y LCR) que fueron colectadas por el médico tratante durante la atención hospitalaria con fines diagnósticos y que cumplieron con la definición de caso.

### Definición de caso

Enfermedad neumocócica invasiva: sospecha clínica de infección por neumococo en pacientes con meningitis, neumonía con derrame o empiema, bacteriemia, artritis séptica y peritonitis bacteriana. Para este estudio, nosotros solo incluimos pacientes con diagnóstico de neumonía adquirida en la comunidad con derrame paraneumónico o empiema y meningitis bacteriana con resultado microbiológico positivo o negativo para neumococo realizado en el hospital o clínica participante.

Neumonía adquirida en la comunidad: infección pulmonar, usualmente en un periodo menor de siete días, con síntomas y signos de compromiso respiratorio (fiebre, taquipnea, hipoxemia, disnea, dolor pleurítico, tos, producción de esputo mucopurulento, signos de consolidación o derrame como matidez, crepitantes, disminución de murmullo vesicular), y evidencia de consolidación pulmonar en la radiografía de tórax.

Derrame paraneumónico: presencia de líquido en el espacio pleural ≥5 cm en la radiografía de tórax o ecografía de tórax con características de exudado [relación proteínas líquido pleural/proteínas sangre >0.5, relación deshidrogenasa láctica (LDH) Líquido pleural/LDH sangre >0.6, LDH líquido pleural >2/3 límite alto normalidad en sangre].

Empiema: Exudado en el espacio pleural con presencia de pus y/o tinción Gram o cultivo positivo en líquido pleural.

Meningitis bacteriana: inflamación de las meninges, con signos y síntomas de fiebre, rigidez de nuca, alteración del nivel de la conciencia y cefalea, así como LCR con leucocitos ≥10 células/mm^3^ a predominio de polimorfonucleares, proteínas >40mg/dL, glucosa <40mg/dL o relación glucosa LCR/sangre <0,5 ^(^^9^.

### Datos clínicos

Se colectaron datos demográficos y clínicos básicos de las historias clínicas, como edad, sexo, día de toma de muestra luego de la hospitalización, uso previo de antibióticos (antes del cultivo) y resultados del cultivo.

### Estudio de laboratorio

Las muestras de LP y LCR colectadas en los hospitales o clínicas fueron transportadas al Laboratorio de Infectología Pediátrica de la Universidad Peruana Cayetano Heredia (UPCH) donde se almacenaron a -20 °C hasta su procesamiento. La extracción de ADN se realizó con 200uL de muestra empleando *Hight pure PCR template Preparation kit®* (Roche, Suiza). Para el diagnóstico molecular de *S. pneumoniae* se estandarizó una qPCR basado en *SYBR Green* (*SsoAdvanced* ^*TM*^ *Universal SYBR*® *Green Supermix*, BioRad) empleando cebadores que amplifican el gen *lytA* que codifica la autolisina en *S. pneumoniae*, la cual es una metodología previamente descrita y validada en la literatura y no forma parte de un kit comercial para la detección de este patógeno ^10^^,^^11^. Se empleó el equipo *CFX96TM Real-Time System* (Bio-Rad®, USA) con las condiciones de desnaturalización inicial de 95°C por 2 min, con 40 ciclos de amplificación con desnaturalización de 95°C por 15 seg., hibridación a 60°C por 30 seg y extensión final con 95 °C 30 seg, además de una curva melting entre 70 - 95 °C con incremento de 0,5 a cada 0,5 seg. En las muestras positivas al gen *lytA* se realizó la serotipificación de neumococo mediante un sistema de PCR multiplex secuencial convencional desarrollado por el Streptococcus Laboratory (StrepLab) del CDC-USA ^12^.

Para poder determinar el límite de detección de la qPCR, se empleó la cepa estándar de *S. pneumoniae* serotipo 2 (D39/NCTC7466). Se realizaron diluciones seriadas en factor de 10 en el rango de 1,2 x 10^6^ a 1,2 x 10^0^ copias de genoma/uL. Se determinó el valor de eficiencia de la prueba generando una regresión lineal evaluando el valor de la pendiente, coeficiente de correlación de linealidad (R^2^) y eficiencia de amplificación.

### Análisis estadístico

La descripción de las características y comparación de frecuencias fueron realizadas mediante la prueba de Chi cuadrado y Exacta de Fisher en el programa estadístico Stata/SE v.18.0. Se consideró p<0,05 como significativo.

### Aspectos éticos

El estudio fue registrado en el Sistema Descentralizado de Información y Seguimiento a la Investigación (SIDISI 100703) y evaluado por el Comité Institucional de Ética de la Universidad Peruana Cayetano Heredia (constancia 062-02-22) y por cada hospital, y clínica participante. Se solicitó consentimiento informado a los pacientes o padres de pacientes pediátricos para usar remanentes de muestras de LP y LCR previamente tomadas por el médico tratante para fines diagnósticos.

## HALLAZGOS

Se analizaron un total de 71 muestras de líquidos (51 de LP y 20 de LCR) de pacientes con sospecha de ENI, según la definición de caso. De estos pacientes, 53,5% fueron de sexo masculino con una mediana de edad de 3 años (RIC: 1 - 9), el 93,9% de los casos fueron en población pediátrica. El 76,6% de las muestras fueron colectadas en los primeros 7 días de la hospitalización del paciente y 63,6% recibió antibióticos previos al cultivo (Tabla 1).

**Tabla 1:** Características de pacientes hospitalizados con meningitis o derrame pleural (N=71).

Características		n (%)
Sexo, masculino		38/71 (53,5)
Edad (meses) ^a^		3 (1 - 9)
Grupo etario		
	Lactantes (<2 años)	18 (25,7)
	Pre-escolares (2 - <6 años)	28 (40,0)
	Escolares (6 - <18 años)	19 (27,2)
	Adultos (≥18 años)	5 (7,1)
Días de la toma de muestra desde la hospitalización		
	0 - ≤3	32 (50,0)
	>3 - ≤7	17 (26,6)
	>7	15 (23,4)
Recibió antibiótico previo al cultivo		42/66 (63,6)
Tipo de muestra		
	Líquido pleural	51 (71,8)
	Líquido cefalorraquídeo	20 (28,2)
Resultado de cultivo		
	*Streptococcus pneumoniae*	28 (39,4)
	Negativo	38 (53,5)
	Otro ^b^	5 (7,1)
qPCR *lytA,* positivo		51/71 (71,8)
*Cycle threshold* (CT)		
	<25	23 (45,1)
	≥25 - <30	10 (19,6)
	≥30	18 (35,3)
Serotipos ^c^		
	No determinado (baja carga)	28
	19A	13
	23A	2
	Serogrupo 24	2
	10A	1
	Serogrupo 6	1
	No tipificable	4

a Mediana (rango intercuartilico).

b *Staphylococcus aureus, Streptococcus grupo* B, S*taphylococcus epidermidis.*

c Serotipo determinado por PCR multiplex secuencial.

El límite de detección de la qPCR fue de 1.2 x 10^1^ copias del genoma/uL, con 40 ciclos de amplificación y no se observaron inespecificidades pasado este ciclo de amplificación. El qPCR presentó un valor de eficiencia de amplificación de 102,9%, y temperatura melting del gen *lytA* de 80.0 ± 0.50 °C (Figura 1).

**Figura 1:** Curva de concentración de *S. pneumoniae* mediante qPCR. A. La evaluación de la qPCRempleando distintas concentraciones de ADN mostra-ron que el límite de detección es de 1,2 x 10^1^ copias del genoma/uL, B. Las curvas melting del ADN extraído de la cepa patrón se superponen, mostrando la reproducibilidad de la qPCR, C. La qPCR mostró una eficiencia de 102,9% para la detección del gen *lyt*A con un coeficiente de correlación de 1,0.

Del total de muestras positivas por qPCR, 23 (45,1%) presentaron un umbral de amplificación o *Cycle threshold* (Ct) <25, es decir una carga bacteriana alta; en estas se pudo determinar el serotipo, siendo el más frecuente el 19A, presente en 13 muestras (25,5% del total) (Tabla 1). En el resto de las muestras, no se logró determinar el serotipo por presentar baja cargar bacteriana (CT≥25).

Mediante qPCR se detectó *S. pneumoniae* en 51 (71,8%) de las muestras analizadas, incluyendo 78,4% (40/51) en LP y 55.0% (11/20) en LCR (Tabla 2). De estas muestras, solo 28 (39,4%) fueron positivas para *S. pneumoniae* por cultivo. Por otro lado, de las 38 muestras negativas por cultivo, se detectó *S. pneumoniae* en 20 de ellas (52,6%) mediante qPCR. En 3 de 5 muestras en las que se aisló otro patógeno por cultivo, se identificó neumococo por qPCR, sugiriendo coinfección. La detección de neumococo fue mayor cuando la muestra se colectó en los primeros días de hospitalización, sin embargo, la diferencia no fue significativa con muestras tardías. En 42 muestras colectadas luego del inicio de antibióticos, la detección de neumococo fue de 20 (47,6%) por cultivo vs. 33 (78,6%) por qPCR.

**Tabla 2:** Resultado de qPCR para diagnóstico de *S. pneumoniae* en líquidos normalmente estériles según características de la muestra (N=71)^a^.

Características		N	qPCR lytA positivo	Cultivo neumococo positivo
			51/71	28/71
			n (%)	n (%)
qPCR *lytA*				
	Negativo	20	0 (0,0)	0 (0,0)
	Positivo	51	51 (71,8)	28 (54,9)
Diagnóstico por cultivo				
	Negativo	38	20 (52,6)	0 (0,0)
	*S. pneumoniae*	28	28 (100,0)	28 (39,4)
	Otro ^b^	5	3 (60,0)	0 (0,0)
Tipo de líquido				
	Líquido pleural	51	40 (78,4)	21 (41,2)
	Líquido cefalorraquídeo	20	11 (55,0)	7 (35,0)
Días de la toma de muestra desde la hospitalización				
	0 - ≤3	32	26 (81,3)	16 (50,0)
	>3 - ≤7	17	13 (76,5)	7 (41,2)
	>7	15	9 (60,0)	4 (26,7)
Recibió antibiótico previo al cultivo				
	No	24	15 (62,5)	7 (29,2)
	Sí	42	33 (78,6)	20 (47,6)

a Algunos variables pueden sumar menos de 71 por datos faltantes.

b *Staphylococcus aureus, Streptococcus* grupo B, *Staphylococcus epidermidis.*

## DISCUSIÓN

Este estudio encontró que la detección de neumococo en líquidos normalmente estériles en pacientes con sospecha de ENI aumentó de 39,4% por cultivo a 71,8% por qPCR. La positividad fue de 78,4% en LP y 55,0% en LCR. En general la positividad fue mayor cuando la muestra se tomó en los primeros tres días de la hospitalización (81,3%). El uso previo de antibióticos no afectó la detección de neumococo mediante la qPCR.

Un dato relevante a destacar es que el 92,8% de las muestras provienen de pacientes pediátricos. A más de diez años de la introducción de las vacunas conjugadas neumocócicas (PCV, en inglés) en el plan nacional de inmunización de Perú, se ha observado una reducción en los casos de ENI ^13^. Sin embargo, estos datos indican que la población pediátrica sigue siendo la más afectada por este patógeno.

Múltiples estudios reportan la capacidad de las técnicas moleculares para mejorar la detección de patógenos en comparación con las técnicas microbiológicas empleando muestras de líquidos normalmente estériles. En el caso de pacientes con meningitis, existen reportes como en Sao Paulo, Brasil al emplear qPCR, la positividad de *S. pneumoniae* aumentó del 9,9% al 14,4% en 263 muestras de LCR ^14^. Un incremento similar en la positividad se reportó en un estudio realizado en pacientes mexicanos, donde aumentó del 0,9% al 5,1% en 512 muestras de LCR ^5^. En pacientes con sospecha de meningitis en Marruecos, el uso de qPCR aumentó la positividad del 11% al 19% ^15^. En un estudio en Fiji en 17 pacientes con meningitis neumocócica confirmada por varios métodos (tinción gram, cultivo, aglutinación de látex y qPCR), la positividad por cultivo fue de 41,0% (7/17) mientras que por qPCR la positividad fue de 100,0% (16/16) ^16^. Asimismo, en un estudio realizado en Egipto, se encontró que de 50 muestras de LCR recolectadas, 28% fueron positivas a *S. pneumoniae* mediante microbiología y 52% empleando qPCR ^17^.

 Con respectó a la detección de *S. pneumoniae* en muestras de líquido pleural, este estudio obtuvo un 41,2% de positividad mediante microbiología y un 78,4% mediante qPCR. En un estudio donde se evaluó 60 líquidos pleurales confirmados con infección neumocócica a través de 16S y/o PCR, solo 6 (10%) tuvieron un cultivo positivo, mientras que 54 (90%) fueron positivos por PCR ^18^. De manera similar, en un estudio cuasiexperimental en Estados Unidos, donde se evaluaron niños con diagnóstico de neumonías complicadas antes y después de la implementación de la detección de *S. pneumoniae* por qPCR en líquido pleural, se observó que la detección de *S. pneumoniae* aumentó en un 34,5% tras la implementación de la qPCR, la cual complementa al cultivo microbiológico utilizado previamente en dicho estudio ^8^. Estos hallazgos sugieren que la mejora en la detección mediante PCR frente al cultivo microbiológico no está relacionada con un solo tipo de muestra biológica.

El tiempo de hospitalización y el consumo de antibióticos previo a la toma de muestra no estuvieron asociados a menor positividad de la qPCR para diagnóstico de *S. pneumoniae*. En contraste, en un estudio transversal realizado en 4676 pacientes pediátricos y adultos con diagnóstico de neumonías adquiridas en la comunidad, reportó que tanto en hemocultivos como en cultivos empleando esputo, la detección de bacterias fue 2,6% (p<0,01) y 23,2% (p<0,01) mayor cuando la muestras fueron colectadas previo al uso del antibiótico. Sin embargo, esto no afectó ni a la detección por PCR ni antigenuria ^19^. Estos informes señalan que el uso de terapia empírica con antibióticos desde el ingreso hospitalario en casos de meningitis y neumonía podría afectar el cultivo de microorganismos mediante métodos microbiológicos estándares.

Una de las limitaciones de este estudio fue que solo se empleó el gen *lyta* como diana para la detección de *S. pneumoniae*. Este gen también puede encontrarse en otras bacterias como *Streptococcus pseudoneumoniae* y estreptococos del grupo *mitis*^20^. Las nuevas recomendaciones sugieren la combinación de genes como *plyA* o *psaA* para la detección de neumococo mediante qPCR ^18^. Sin embargo, esta recomendación es para estudios en portadores, debido a que *S. pneumoniae* comparte el lugar de colonización con otros microorganismos. A pesar de esto, la qPCR que detecta el gen *lytA* sigue siendo uno de los más sensibles para la detección de enfermedad neumocócica invasiva ^5^.

En conclusión, la estandarización de esta qPCR con un límite de detección de 1,2x10^1^ copias del genoma/uL, es una herramienta útil para mejorar la detección de neumococo en líquidos normalmente estériles con sospecha de ENI. La qPCR representa una alternativa rápida en comparación a los tres días de espera para el resultado del cultivo, con lo cual se puede instaurar tempranamente una adecuada terapia antibiótica. Esta técnica resulta además útil para los casos de uso previo de antibióticos.
